# The use of a blood conservation device to reduce red blood cell transfusion requirements: a before and after study

**DOI:** 10.1186/cc8859

**Published:** 2010-01-27

**Authors:** Amartya Mukhopadhyay, Hwee S Yip, Dimple Prabhuswamy, Yiong H Chan, Jason Phua, Tow K Lim, Patricia Leong

**Affiliations:** 1 Department of Medicine, National University Hospital, National University Health System, 5 Lower Kent Ridge Road, Singapore 119074, Singapore; 2 Biostatistics Unit, Yong Loo Lin School of Medicine, National University of Singapore, 10 Medical Drive, Level 2, Block MD11, Singapore 117597, Singapore; 3 Medical Intensive Care Unit, National University Hospital, National University Health System, 5 Lower Kent Ridge Road, Singapore 119074, Singapore

## Abstract

**Introduction:**

Anaemia and the associated need for packed red blood cell (PRBC) transfusions are common in patients admitted to the intensive care unit (ICU). Among many causes, blood losses from repeated diagnostic tests are contributory.

**Methods:**

This is a before and after study in a medical ICU of a university hospital. We used a closed blood conservation device (Venous Arterial blood Management Protection, VAMP, Edwards Lifesciences, Irvine, CA, USA) to decrease PRBC transfusion requirements. We included all adult (≥18 years) patients admitted to the ICU with indwelling arterial catheters, who were expected to stay more than 24 hours and were not admitted for active gastrointestinal or any other bleeding. We collected data for six months without VAMP (control group) immediately followed by nine months (active group) with VAMP. A restrictive transfusion strategy in which clinicians were strongly discouraged from any routine transfusions when haemoglobin (Hb) levels were above 7.5 g/dL was adopted during both periods.

**Results:**

Eighty (mean age 61.6 years, 49 male) and 170 patients (mean age 60.5 years, 101 male) were included in the control and active groups respectively. The groups were comparable for age, gender, Acute Physiology and Chronic Health Evaluation (APACHE) II score, need for renal replacement therapy, length of stay, and Hb levels on discharge and at transfusion. The control group had higher Hb levels on admission (12.4 ± 2.5 vs. 11.58 ± 2.8 gm/dL, *P *= 0.02). Use of a blood conservation device was significantly associated with decreased requirements for PRBC transfusion (control group 0.131 unit vs. active group 0.068 unit PRBC/patient/day, *P *= 0.02) on multiple linear regression analysis. The control group also had a greater decline in Hb levels (2.13 ± 2.32 vs. 1.44 ± 2.08 gm/dL, *P *= 0.02) at discharge.

**Conclusions:**

The use of a blood conservation device is associated with 1) reduced PRBC transfusion requirements and 2) a smaller decrease in Hb levels in the ICU.

## Introduction

A significant number of patients in the intensive care unit (ICU) receive packed red blood cell (PRBC) transfusions [1]. Anaemia which affects up to 90% of ICU patients by Day 3 is multifactorial [1]. One such cause is blood loss, up to 17% of which is contributed by repeated blood drawing for diagnostic tests [2,3]. Blood samples may be drawn up to 24 times in a day, resulting in an average blood loss of 41 ml on Day 1 [4]. There is a positive correlation between organ dysfunction and the number of blood draws [2,3,5]. The presence of indwelling central venous or arterial catheters makes blood sampling easier but contributes to iatrogenic anaemia as the first few millilitres of infusate-blood mixture obtained while collecting blood from such catheters are discarded [6-8]. In two large trials, 37 to 44% of patients in ICU received PRBC transfusions [1,5] often at high transfusion thresholds, despite evidence to support a restrictive transfusion practice to keep haemoglobin (Hb) levels in the range of 7 to 9 g/dL [9]. Importantly, PRBC transfusions are associated with adverse effects, including allergic, anaphylactic and haemolytic transfusion reactions, transfusion-related acute lung injury (TRALI), transfusion-associated circulatory overload (TACO), acute respiratory distress syndrome (ARDS), infections, and ventilator-associated pneumonia, all of which lead to significant morbidity and mortality [10-14].

Reduction of the discarded blood volume is possible using a three-way connection [15] or a dedicated blood conservation system [16]. While data exist to show that such devices may reduce the degree of blood loss [17,18] resulting in higher Hb levels [19], no previous study has demonstrated any significant effect of these devices on the amount of blood transfusion. This apparent paradox may be related to the inadequate sample sizes or study design issues including the lack of standardised thresholds for transfusions [20]. The primary objective of the present study is therefore to investigate if the use of a blood conservation device in the presence of a standardised restrictive transfusion practice can reduce the number of units of PRBC transfused. The secondary objective is to investigate if the use of the device is associated with a smaller decrease in Hb levels from ICU admission to discharge.

## Materials and methods

### Study design

This was a before-and-after study conducted in the 12-bed medical ICU of our university hospital. The before-study period included patients from January to June 2008 (control group). The blood conservation device was introduced to the active group at the start of the after period from July 2008 to March 2009 (active group).

### Patients

We included all patients admitted to the ICU who were 1) 18 years and above, 2) expected to stay more than 24 hours and 3) had an indwelling intra-arterial catheter inserted. We excluded patients who 1) were expected to stay less than 24 hours and 2) had active gastrointestinal or other bleeding as the primary cause of ICU admission. Patients were followed up till hospital discharge, death or up to 28 days of ICU stay, whichever was later.

### Device

We used the Venous Arterial blood Management Protection (VAMP) system (Edwards Lifesciences, Irvine, CA, USA) for the active group. This device has been described previously [16]. Briefly, it is attached to the existing arterial catheter. While drawing the samples the flexures of the device are firmly squeezed and a blood volume is slowly drawn into the reservoir over three to five seconds. The shut-off valve just proximal to the reservoir towards the patient's end is then closed. The sample site is cleaned and a syringe with a custom-made cannula (Edwards Lifesciences) is attached. A vacuum tube is attached to the syringe and the required blood sample(s) is drawn. Following the collection of the sample, the syringe with the cannula is removed and the shut-off valve is opened. The device's plunger is then pushed down smoothly and evenly over three to five seconds, until the flexures lock in place in the fully closed position and all fluids have been reinfused into the arterial line. A single device was used for an individual arterial catheter throughout the patient's stay and removed or changed with the arterial catheter.

### Transfusion practice

We employed a restrictive transfusion practice in both the before and after periods of the study [9]. Clinicians were strongly discouraged against any routine transfusion of PRBCs when the Hb level was above 7.5 g/dL, unless there was a physiological need for transfusions (including transfusion as part of resuscitation, preoperatively, or in patients with coronary artery disease). Ultimately, however, the decision to transfuse was left to the discretion of the clinicians.

### Outcomes

The primary outcome was the number of units of PRBC transfused per patient per day of ICU stay. The secondary outcome was the difference between the Hb levels at ICU admission and discharge.

### Data collection

We recorded the following data prospectively: patient demographics, Acute Physiology and Chronic Health Evaluation (APACHE) II score, Hb levels at ICU admission and discharge or death and just before any PRBC transfusion, number of units of PRBC transfused, need for any renal replacement therapy (RRT), ICU length of stay (LOS), and mortality. For the patients who died in ICU, the last Hb before death was recorded.

### Sample size

On a ratio of one control to two active patients, with 80% power and a two-sided test of 5%, 80 controls to 160 patients will provide a statistically significant result for a difference of at least 0.05 unit PRBC/patient/day with a standard deviation of 0.15 units PRBC/patient/day.

### Statistical analysis

We expressed variables as means ± standard deviations and numbers (percentages), and made comparisons using Student's *t*-test and the chi-square test where appropriate. To elucidate the independent predictors of transfusion requirement, the following variables were entered into a linear regression model: age, gender, Hb on admission and just before transfusions, LOS, severity of illness, RRT (duration in hours), and use of the blood conservation device. The same variables were entered into a separate logistic regression model to ascertain the independent predictors of ICU and hospital mortality. We used the statistical software SPSS version 17.0 (SPSS Inc., Chicago, IL, USA).

The study was approved by our Institutional Review Board and Ethics committee. Informed consent was obtained in the active group. Requirement of consent was waived for the control group.

## Results

**Figure 1:**
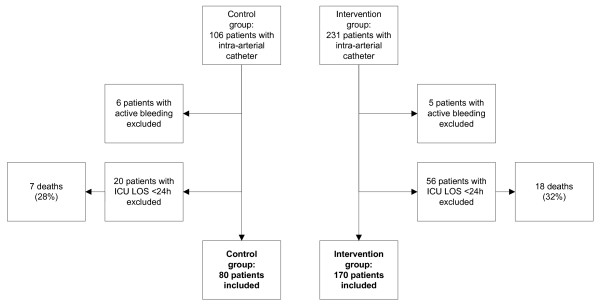
**Patient enrollment**. LOS = length of stay.

There were 80 patients in the control group and 170 patients in the active group (Figure 1). There were no significant differences in age, gender, APACHE II score, and percentage of patients requiring RRT in the two groups (Table 1). There were no complications associated with VAMP device.

**Table 1:** Baseline characteristics

Baseline characteristics	Control group: without blood conservation device (n = 80)	Active group: with blood conservation device (n = 170)	*P-value*
**Underlying aetiology**			
Sepsis			0.71
Pulmonary	28 (35%)	65 (38%)	
Extra-pulmonary	18 (22.5%)	31 (18%)	
Airway disease	7 (9%)	18 (10.5%)	0.82
Neurological	6 (7.5%)	11 (6.5%)	0.79
Renal failure, Metabolic acidosis	5 (6%)	11 (6.5%)	1.0
Acute Pulmonary Odema	5 (6%)	12 (7%)	1.0
Others	11 (14%)	23 (13.5%)	1.0

**Age **(years)	61.6 ± 18.3	60.5 ± 15.5	0.62

**Male/Female**	49/31	101/69	0.78

**APACHE II score**	18.6 ± 7	21.24 ± 7.8	0.09

**RRT **(%)	21	24	0.4

**Hb on admission **(g/dL)	12.4 ± 2.5	11.58 ± 2.8	0.02

APACHE = Acute Physiology and Chronic Health Evaluation, RRT = renal replacement therapy, Hb = haemoglobin

### Transfusion and Hb levels

Although baseline Hb levels at admission were significantly lower in the active group compared to the control group, the active group required less PRBC transfusion (0.068 vs. 0.131 units/patient/day) (Table 2). Analysis by the linear regression model showed that the use of a blood conservation device was independently associated with lower PRBC requirements (*P *= 0.02, Table 3).

**Table 2:** Transfusion and haemoglobin levels

	Control group: without blood conservation device	Active group: with blood conservation device	*P*-value
	
All patients, *n*	80	170	
PRBC transfusion (unit/patient/day)	0.131	0.068	0.02*

Hb on admission (g/dL)	12.4 ± 2.5	11.58 ± 2.8	0.02

Hb on discharge (g/dL)	10.2 ± 1.8	10.1 ± 2	0.6

Loss of Hb (g/dL)	2.13 ± 2.32	1.44 ± 2.08	0.02

			

Patients with transfusion, *n *(%)	17 (21.3)	52 (30.6)	0.12

Hb at transfusion (g/dL)	7.1 ± 0.85	7.25 ± 1.1	0.5

Transfusion above Hb of 7.5 g/dL (%)	23.8	29.7	0.3

Patients without transfusion, *n *(%)	63(78.7)	118 (69.4)	0.12

Loss of Hb (g/dL)	1.97 ± 2.00	1.83 ± 1.77	0.6

PRBC = packed red blood cell, Hb = haemoglobin

* Adjusted for the variables in Table 3

**Table 3:** Adjusted estimates for control vs active on PRBC transfusion requirements and mortality outcomes

	PRBC transfusion (unit/patient/day)		ICU Mortality		Hospital Mortality	
	**B Estimate (95% CI)**	***P*-value**	**OR (95% CI)**	***P*-value**	**OR (95% CI)**	***P*-value**

Control vs Active	0.063 (0.010, 0.116)	0.02	0.34 (0.19, 0.60)	< 0.001	0.36 (0.20, 0.63)	< 0.001

Age (years)	-0.003 (-0.005, -0.002)	< 0.001	0.99 (0.97, 1.01)	0.268	0.99 (0.98, 1.01)	0.608

Male vs Female	-0.035 (-0.085, 0.014)	0.158	1.1 (0.61, 1.9)	0.782	1.2 (0.7, 2.1)	0.502

APACHE II score	0.003 (-0.001, 0.007)	0.067	1.01 (0.97, 1.05)	0.589	1.004 (0.97, 1.04)	0.82

Hb before transfusion (g/dL)	0.050 (0.041, 0.059)	< 0.001	0.95 (0.86,1.05)	0.337	0.98 (0.89, 1.08)	0.647

Hb on admission (g/dL)	-0.001 (-0.011, 0.009)	0.842	0.92 (0.82,1.03)	0.157	0.95 (0.85, 1.06)	0.331

ICU LOS (days)	-0.006 (-0.009, -0.002)	0.001	0.99 (0.95, 1.03)	0.627	0.99 (0.95, 1.03)	0.616

RRT (Duration, hours)	0.0003 (-0.0001, 0.001)	0.197	1.004 (0.99, 1.01)	0.176	1.001 (0.99, 1.007)	0.646

Hb = haemoglobin, ICU = intensive care unit, LOS = length of stay, APACHE = Acute Physiology and Chronic Health Evaluation, RRT = renal replacement therapy

The Hb on admission was significantly higher in the control group (12.4 ± 2.5 vs. 11.58 ± 2.8, *P *= 0.02) but were similar at discharge in both groups. Correspondingly, there was a smaller drop in Hb levels between admission and discharge in the active group than in the control group (mean 1.44 vs. 2.13 g/dL, *P *= 0.02, Table 2).

Seventeen (21.3%) patients in the control group received 62 units of PRBC over 42 episodes of transfusion and 52 (30.6%) patients in the active group received 129 units of PRBC over 84 episodes. The Hb level at transfusion was above the suggested threshold in 10/42 (23.8%, range 7.6 to 9.2 g/dL) episodes in the control group and 25/84 episodes (29.7%, range 7.6 to 11 g/dL) in the active group (*P *= 0.3, Table 2).

Sixty-three patients in the control and 118 patients in the active group did not receive any packed cell transfusions (Table 2). There was no significant difference in the change in Hb levels from admission to discharge between these groups.

### Mortality and length of stay

ICU (control group 31/80, 38% vs. active group 37/170, 21%, *P *= 0.001) and hospital (control group 43/80, 53% vs. active group 51/170, 30%, *P *= 0.001) mortality were significantly higher in the control group. Even after adjusting for other variables including gender, age, RRT, Hb on admission and at transfusion, LOS and APACHE II score, mortality in the active group remained significantly less (Table 3). The ICU LOS was similar in both groups (control group 6.6 ± 4.8 vs active group 8.3 ± 8.1 days, *P *= 0.09).

## Discussion

In the present study, patients using a blood conservation device had a 48% reduction in PRBC transfusion requirements. This was not observed in previous studies using similar devices. The device was also associated with a smaller decrease in Hb levels between ICU admission and discharge.

Use of blood conservation devices has been studied previously. Three-way stopcock and syringes can be used to preserve the discarded blood-infusate [4]. Silver MJ et al [19] showed that the blood samples obtained with the blood-conserving arterial line were free of haemodilution or heparin contamination. In a small randomised control trial (RCT), Peruzzi WT et al [19] showed that the conservation group had better preservation of Hb with less volume of blood being discarded. However, the decrease in the transfusion requirements was not significant. Such devices were also found to be free of microbial contaminations [21].

Despite their potential benefits, blood conservation devices are rarely used. In a survey of members of the Society of Critical Care Medicine, most agreed that such devices could be very useful in preventing anaemia [4]. Another survey found that such devices were used in only 18.4% of adult ICUs in England and Wales [22]. One reason for such a paradox is the lack of convincing data on the effect of these devices on transfusion requirements. Encouragingly, findings of the present study strongly suggest that such devices do indeed reduce PRBC transfusion.

Determination of a transfusion threshold or trigger in the ICU has been challenging. Due to the adoption of a restrictive transfusion practice [9] in our ICU, only 27.6% of our patient cohort received PRBC transfusions, which is lower than in previous studies [1,5]. This is reflected in the similar Hb levels at transfusion in both the control and active (7.1 ± 0.85 vs 7.25 ± 1.1 g/dL) groups. It is likely that concurrent application of the restrictive transfusion practice where transfusion triggers are not individualised but guided, allowed demonstration of the effect of the blood conservation device on transfusion requirements. This notwithstanding, 23.8% and 29.7% patients in the control and active group respectively did receive transfusions above the suggested threshold (Table 2). In addition, a relatively smaller number of patients in the control group (control 17/80, 21.3% vs active 52/170, 30.6%) received a larger number of PRBC transfusions (control 62 units vs active 129 units of PRBC, table 2). This suggests that multiple transfusions of the same patients occurred in the control group.

In our study, the control group had a greater loss of Hb; this finding is consistent with those of previous studies [15,18,19]. Patients in the control group had higher Hb levels on admission but similar Hb levels at discharge from ICU. There was also a numerical, though not statistically significant, trend toward better preservation of the Hb at discharge in the group without transfusion (Table 2).

Patients with the blood conservation device had a significantly lower ICU and hospital mortality. While these findings must be interpreted with caution since the present study was not an RCT and mortality was not our primary or secondary end-point, they do suggest a protective effect of reduced transfusion. Indeed, blood transfusion was associated with higher mortality in both the CRIT and ABC trials [1,5]. Nonetheless, it should be noted that among the patients who stayed in the ICU for less than 24 hours, a larger number of patients died in the active group which may have contributed to the improved mortality in the remaining patients.

We acknowledge the limitations of our study. First, this was a before-and-after study and given the limitations of historical control study, the results of our study need to be confirmed with prospective RCT. Second, physicians and nurses were not blinded to the device. Nonetheless, we attempted to ensure equal treatment of both groups with the common restrictive transfusion strategy, which was reflected by the similar transfusion thresholds between the two periods. Third, we only included patients admitted to the medical ICU and expected to stay more than 24 hours. Although the largest volume of blood is drawn during the first 24 hours [18], such a short study period may be insufficient to demonstrate any reduction in the PRBC transfusions. A previous study has shown that the higher mean Hb in the blood conservation group was statistically significant only after 9.5 days of ICU stay [19]. Fourth, we excluded patients with active bleeding where transfusion practices may differ. Fifth, we used the VAMP device and it remains to be seen if our findings are applicable to other blood conservation devices.

## Conclusions

Since anaemia is the main reason for transfusion in the ICU, and a blood conservation device is associated with better preservation of Hb, it is logical that use of such a device will reduce transfusion requirements. In this before-and-after study, use of a blood conservation device in the presence of a restrictive transfusion practice was indeed associated with a significant reduction in blood transfusion requirements. The significance of this finding is clear given the current worldwide shortage of PRBCs, but extends far beyond apparent cost-benefit ratio and economic savings. PRBC transfusions are associated with significant morbidity and mortality and any reduction in transfusions may eventually improve overall patient outcome. A larger prospective RCT is currently being planned.

## Key messages

• Anaemia is common in critically ill patients admitted to ICU and as a result, large numbers of patients receive blood transfusions.

• Blood transfusions are in short supply, expensive and have deleterious effects on patient outcome.

• Previous studies have shown that by preserving the discarded volume of blood from indwelling arterial or central line catheters, blood conservation devices can improve anaemia (Hb).

• The present study shows that with restrictive transfusion practice, blood conservation devices can reduce blood transfusion requirements.

## Abbreviations

APACHE: Acute Physiology and Chronic Health Evaluation; ARDS: acute respiratory distress syndrome; Hb: haemoglobin; ICU: intensive care unit; LOS: length of stay; MICU: medical intensive care unit; PRBC: packed red blood cell; RRT: renal replacement therapy; TACO: transfusion-associated circulatory overload; TRALI: transfusion-related acute lung injury; VAMP: Venous Arterial blood Management Protection.

## Competing interests

The authors declare that they have no competing interests.

## Authors' contributions

AM was involved in study conception, design, securing fund, analysis and manuscript drafting. YHS was involved in institutional review board, ethics committee approval and manuscript drafting. DP was involved in data collection. CYH was involved in statistical analysis. JP and LTK were involved in manuscript drafting and PL was involved in running the project.

## Authors' information

AM (medical intensivist) is currently the clinical director of the medical ICU of the author's hospital. YHS is a registrar in the division of respiratory and critical care of the author's hospital. DP is a research assistant. CYH is the head of the biostatistics unit of the Yong Loo Lin School of Medicine, National University of Singapore. JP (medical intensivist) is a consultant in the division of respiratory and critical care medicine of the author's hospital. LTK is the head of the division of respiratory and critical care medicine of the author's hospital. PL is the nurse clinician of the medical ICU of the author's hospital.
